# The Transcription Factor EGR2 Plays a Central Role in the Expansion and Function of TCRαβ ^+^ CD4 ^−^ CD8 ^−^ Double Negative T Cells in lpr Lupus Mice

**DOI:** 10.1111/imm.70162

**Published:** 2026-06-21

**Authors:** Rujuan Dai, Zhuang Wang, Bettina Heid, Christopher M. Reilly, S. Ansar Ahmed

**Affiliations:** ^1^ Department of Biomedical Sciences and Pathobiology Virginia‐Maryland College of Veterinary Medicine (VMCVM), Virginia Tech Blacksburg Virginia USA; ^2^ Via Virginia College of Osteopathic Medicine Blacksburg Virginia USA

## Abstract

TCRαβ^+^CD4^−^CD8^−^ double negative T (TCRβ^+^DNT) cells are highly accumulated in human and murine lupus, although the origin and function of TCRβ^+^DNT cells remains largely elusive and controversy. Remarkably, conditional deletion of *Egr2* in lymphocytes significantly reduced TCRβ^+^NK1.1^−^ DNT cells, the dominant DNT type in B6/*lpr* mice. TCRβ^+^DNT cells of B6/*lpr* mice surprisingly had a lower expression of IFNγ, IL‐17, and IL‐10 compared to normal B6 mice, which was likely due to high CD138 expression in the TCRβ^+^DNT cells of B6/*lpr* mice. Conditionally deleting *Egr2* in B6/*lpr* mice increased IFNγ and IL‐10, but not IL‐17 in TCRβ^+^DNT cells. *Egr2* deletion suppressed IL‐17 expression in TCRγδ^+^DNT cells, the major IL‐17‐expressing DNT cells in B6/*lpr* mice. The scRNA‐seq analysis of DNT cells from *Egr2* ^*−*/−^B6/*lpr* (CD2‐Cre*Egr2* ^*−*/−^B6/*lpr*, EK) and control *Egr2* ^*fl/fl*^B6/*lpr* (EF) mice revealed a striking segregation of DNT clusters with distinct transcriptional profiles, identified as EK_dominant and EF_dominant DNT clusters. *Egr2* deletion in B6/*lpr* mice seemingly normalised Helios, Ly6C, and Ly6A/E expression to levels similar to B6 mice, implying that inhibition of EGR2 can effectively correct the abnormalities in DNT cells of B6/*lpr* mice. Further, we found that conditional *Egr2* deletion reduced TCRβ^+^DN thymocytes in B6/*lpr* mice and suppressed DNT‐cell generation from in vitro cultured splenic CD8^+^ T cells, suggesting that EGR2 might promote TCRβ^+^DNT cells in *lpr* mice at both the thymic and peripheral stages. Together, this study is the first to reveal a critical role of EGR2 in regulating the expansion, heterogeneity, and function of TCRβ^+^DNT cells in lupus mice.

## Graphical abstract

The highly expanded TCRβ^+^ DNT cells in autoimmune‐prone B6/*lpr* mice are phenotypically and functionally different from the TCRβ^+^DNT cells of normal B6 mice. Conditional *Egr2* deletion in B6/*lpr* mice not only reduces TCRβ^+^ DNT cell numbers but also tends to correct the phenotypic and functional abnormalities of DNT cells in B6/*lpr* mice. In addition, *Egr2* deletion suppressed IL‐17 in TCRγδ^+^DNT cells, the major IL‐17‐producing DNT cells in B6/*lpr* mice.

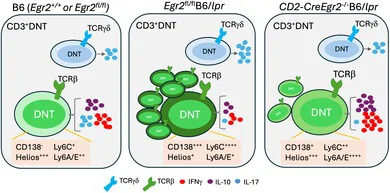

## 1 Introduction

CD3^+^CD4^−^CD8^−^ double negative T (DNT) cells are a rare subset of T lymphocytes that express T cell receptor (TCR) αβ or TCRγδ, but lack CD4 and CD8 co‐receptors. In normal humans and mice, TCRαβ (TCRβ)^+^DNT cells represent only approximately 1%–3% of T cells [1, 2]. TCRβ^+^DNT have gained increased interest due to their dual capability to function as pathogenic or immunoregulatory cells in different pathological and physiological conditions [3, 4, 5, 6]. Despite significant efforts, the origin and the molecular regulation of TCRβ^+^DNT‐cell development and function remain poorly understood.

In lupus and multiple other autoimmune diseases, TCRβ^+^DNT cells are markedly expanded and display a pathogenic function by producing inflammatory cytokines such as IFNγ and IL‐17 and/or by promoting B cells to produce IgG and pathogenic anti‐dsDNA autoantibodies [5, 6, 7, 8, 9]. Nevertheless, TCRβ^+^DNT cells have also been reported to play an immune regulatory role in preventing graft‐versus‐host disease (GVHD), allograft rejection, and non‐obese diabetes (NOD), potentially through the production of immune suppressive cytokines IL‐10, directly killing effector T cells, or by other unknown mechanisms [10, 11, 12]. The diverse functions of TCRβ^+^DNT cells reported across various studies may be attributed to their heterogeneity. By performing single‐cell RNA sequencing (scRNA‐seq) of naïve TCRβ^+^NK1.1^−^ DNT (nDNT) from normal C57BL/6 (B6) mice, Yang et al. characterised five transcriptionally distinct nDNT subgroups, which exert different functions [13]. Among them, resting DNT (nDNT0) accounts for the majority (about 78%). Following stimulation by anti‐CD3/anti‐CD28 stimulation, the activated DNT cells (aDNTs) are majority cytotoxic DNTs (83.8%), and the rest are proinflammatory DNTs.

Early Growth Response 2 (EGR2) is a transcription factor that plays a vital role in the regulation of T cell development, T cell anergy, and effector T cell responses [14, 15, 16, 17]. Increased *Egr2* expression has been linked with lupus susceptibility in humans and identified in both human and murine lupus [18, 19]. While conditionally deleting *Egr2* in B6 mice promoted the production of anti‐dsDNA autoantibodies and IL‐17, conditionally deleting *Egr2* in autoimmune‐prone B6/*lpr* mice suppressed anti‐dsDNA autoantibodies and IL‐17 production [15, 20]. This suggests a context‐dependent immune and autoimmune regulatory role of EGR2. Of particular interest, *Egr2* deletion significantly reduced CD3^+^DNT cells in the spleens of both B6/*lpr* and B6 mice [20], revealing a novel central role of EGR2 in regulating DNT‐cell development in both healthy and diseased conditions. In this study, we showed that *Egr2* deletion tended to correct the abnormality of DNT cells in B6/*lpr* mice by suppressing the expansion and altering the phenotypes and functions of TCRβ^+^NK1.1^−^DNT subsets. This study is the first to report a key regulatory role of EGR2 on DNT cells in lupus and provides a new insight into the molecular regulation of DNT cell development under autoinflammation conditions.

## 2 Material and Methods

### 2.1 Mice

Both wild type B6 (*Egr2* ^+/+^, purchased from the Jackson Laboratory (JAX)) and *Egr2*‐floxed B6 mice (*Egr2* ^fl/fl^) were used as B6 controls for *Egr2* ^fl/fl^B6/*lpr* mice. The development and characterisation of *Egr2* ^fl/fl^B6, *Egr2* ^fl/fl^B6/*lpr*, and conditional *Egr2* knockout B6/*lpr* (CD2‐Cre*Egr2* ^−/−^B6/*lpr*, *or Egr2* ^−/−^B6/*lpr*) strains have been reported [20]. The mice were housed in an Association for Assessment and Accreditation of Laboratory Animal Care (AAALAC)‐certified animal facility at the VMCVM, Virginia Tech. All studies and procedures were performed under animal protocols approved by the Institutional Animal Care and Use Committee (IACUC) of Virginia Tech.

### 2.2 Cell Preparation and Flow Cytometry

The single cell suspensions of splenocytes and thymocytes were prepared following previously reported procedures [21, 22, 23]. Flow cytometric analysis was performed to determine specific immune cell subsets, intracellular cytokines, and nuclear antigens as we reported previously [19, 20]. Dead cells were excluded from the analysis with propidium iodide or Zombie Aqua fluorescent dye staining (Biolegend).

### 2.3 Flow Sorting of CD8 ^+^ Single Positive (CD8 ^+^ SP) T Cells and CD3 ^+^ DNT Cells

Since *lpr* mice have a large portion of CD3^+^B220^+^DNT cells in the spleens [24, 25], CD8^+^ T cells were enriched by depleting non‐CD8 cells. Briefly, the freshly‐isolated splenocytes were stained with a cocktail of biotin‐conjugated monoclonal antibodies (Miltenyi Biotech) against CD19, CD11b, CD4, B220, Ter119, CD49B, and CD105, and then incubated with anti‐Biotin microbeads. The labelled cell suspension was applied to a MACS column. The flow through containing enriched CD8^+^ T cells was collected. DNT cells were enriched with a similar approach as for CD8^+^ T cells except that anti‐B220 antibodies were excluded from the cocktail of biotin‐conjugated antibodies. The enriched CD8^+^ T cells and DNT cells were used for sorting CD8^+^ SP and CD3^+^DNT cells. The purity of the sorted CD8^+^ SP and DNT cells was over 99% and the viability was over 98%.

### 2.4 In Vitro Culture of CD8 ^+^ T Cells

The sorted CD8^+^ T cells were adjusted to 1.5 × 10^6^/mL in complete RPMI‐1640 medium (formulated as we previously reported [19, 20]), and stimulated with anti‐CD3 (plate coated at 5 μg/mL, Bio X cell) plus anti‐CD28 (soluble at 5 μg/mL, Bio X cell) for 5 days before flow analysis [26].

### 2.5 RT‐qPCR

Total RNA was prepared from CD3^+^DNT cells with RNeasy Micro kit (Qiagene). RT‐qPCR was performed as we previously reported [19]. The qPCR primer sequences for murine *Cd8a*: forward 5′‐GAT TGG ACT TCG CCT GTG ATA‐3′ and reverse 5′‐GTG GTA GCA GAT GAG AGT GAT G‐3′; for murine *Cd8b1*: forward 5′‐AAT GTG AAG CCA GAG GAC AG‐3′ and reverse 5′‐GCA GTT GTA GGA AGG ACA TCA‐3′.

### 2.6 scRNA‐Seq Library Preparation, Sequencing, and Data Analysis

The sorted CD3^+^DNT cells were fixed with Evercode Cell Fixation Kit for library preparation with Evercode WT V2 kit (Parse Bioscience) according to manufacturer instructions. The pre‐made library DNA was sent to Novogene for sequencing using NovaSeq X Plus (PE 150‐10B) platform with a target of 60K reads per cell.

The raw sequencing data were analysed using Parse Biosciences Trailmaker web service. The raw sequencing reads were mapped to mouse reference genome (GRCm39, mm39). The output expression matrix was further analysed with R package Seurat (version 4.3.0) [27]. Low quality cells with fewer than 300 detected genes or with higher than 10% mitochondrial gene content were excluded from further analysis. Doublets were detected by scDoublets and removed. The filtered expression matrices were normalised with NormalizeData function, and the top 2000 highly variable genes were identified with FindVariableFeatures, then scaled and centred with ScaleData. Further cell clustering was performed using FindNeighbors and FindClusters with the first 8 principal components. UMAP visualisation is conducted using RunUMAP function based on selected principal components. The differentially expressed genes were identified by FindAllMakers and FindMarkers functions. Pathway enrichment analysis of differentially expressed genes was performed with clusterProfiler (Version 4.2.2) [28]. The raw and processed data were deposited into the Gene Expression Omnibus (GEO) database with accession number GSE306407.

### 2.7 Statistical Analysis

GraphPad Prism was used to generate summary data graphs. The outliers were identified with the embedded ROUT method and excluded from the summary graphs. One‐way ANOVA with Tukey–Kramer all pair's comparisons and two‐tailed, unpaired Student's *t*‐tests were performed for statistical analysis.

## 3 Results

### 3.1 *Egr2* Deletion in B6/*Lpr* Mice Significantly Reduces TCRβ ^+^ NK1.1^−^ DNT Cells and Rebalances DNT Subpopulations in Splenic CD3 ^+^ DNT Cells

**FIGURE 1:**
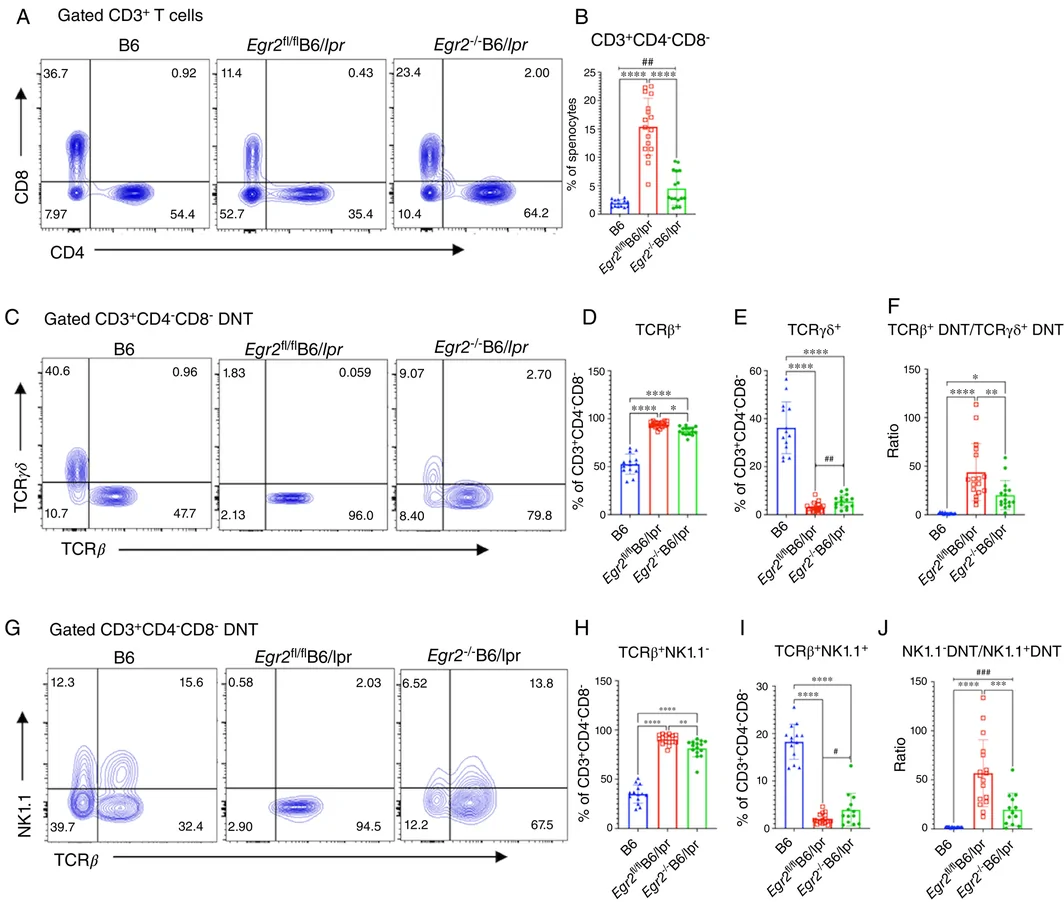
*Egr2* deletion in B6/*lpr* mice significantly reduces TCR𝛽^+^NK1.1^−^ DNT cells and partially restores the proportional distribution of TCR𝛽^+^ DNT, TCRγδ^+^ DNT, and DN‐NKT subpopulations within CD3^+^ DNT compartments. Splenocytes were isolated from B6 mice (either *Egr2* ^+/+^ or *Egr2* ^fl/fl^) at 11–36 weeks old, *Egr2* ^fl/fl^B6/*lpr* and *Egr2* ^−/−^B6/*lpr* mice at 15–30 weeks old (*n* ≥ 14 each) for flow. Both females and males were included in the assay. (A&B) Representative flow plots and summary graph show the percentage of CD3^+^DNT cells in the spleens of B6, *Egr2* ^fl/fl^B6/*lpr*, and *Egr2* ^−/−^B6/*lpr* mice. (C–E) Representative flow plots and summary graphs showing the percentage of TCR𝛽^+^ cells, TCRγδ^+^ cells in the gated CD3^+^DNT cells. (F) Summary plot of the ratio of TCR𝛽^+^ DNT to TCRγδ^+^ DNT cells. (G–I) Representative flow plots and summary graphs show the percentages of TCR𝛽^+^NK1.1^−^ and TCR𝛽^+^NK1.1^+^ cells within the gated CD3^+^DNT cells. (J) Summary plot of the ratio of TCR𝛽^+^NK1.1^−^ DNT to TCR𝛽^+^NK1.1^+^ DNT cells. Data were obtained from four independent experiments. One‐way ANOVA for multiple groups comparison (displayed with * with brackets) and/or unpaired Student's *t* tests for two groups comparison (displayed with # with line) were performed; * and # *p* < 0.05; **p < 0.01; *** and ###*p* < 0.001; ****p < 0.0001.

We observed a remarkable expansion of CD3^+^DNT cells in the spleen of B6/*lpr* mice compared to normal B6 mice (Figure 1A), in agreement with the previous reports [24, 29]. Consistent with our earlier observations [20], *Egr2* deletion in B6/*lpr* mice significantly reduced splenic CD3^+^DNT cells (Figure 1A,B). While the CD3^+^DNT cells in B6 mice comprised both TCRβ^+^ and TCRγδ^+^ cells, almost all the splenic CD3^+^DNT cells in B6/*lpr* mice were TCRβ^+^ cells (Figure 1C–E). The ratio of TCRβ^+^DNT to TCRγδ^+^DNT cells in B6 mice was 1.66 ± 0.79, which was increased to 43.82 ± 29.44 in B6/*lpr* mice (Figure 1F). *Egr2* deletion significantly reduced the percentage of TCRβ^+^ cells and increased the percentage of TCRγδ^+^ cells in the gated CD3^+^DNT cells, which brought the ratio down to 20.26 ± 15 in *Egr2* ^−/−^B6/*lpr* mice (Figure 1D–F).

Within TCRβ^+^DNT group, the expression of NK1.1 distinguishes two functionally distinct subsets: NK1.1^+^DNT (DN‐NKT) and NK1.1^−^DNT. These TCRβ^+^NK1.1^−^DNT cells are the population that expands in autoimmune diseases such as lupus [4, 5]. In B6 mice, approximately 20% of CD3^+^DNT cells were DN‐NKT, but in B6/*lpr* mice, almost all the CD3^+^DNT cells were TCRβ^+^NK1.1^−^ (Figure 1G–I). The ratio of TCRβ^+^NK1.1^−^DNT to DN‐NKT in B6 mice was 1.97 ± 0.33, which was increased to 56.88 ± 34.04 in B6/*lpr* mice. *Egr2* deletion in B6/*lpr* mice significantly reduced TCRβ^+^NK1.1^−^ DNT cells and brought the ratio down to 19.72 ± 16.75 (Figure 1J). Together, our data demonstrated that *Egr2* deletion drastically reduced TCRβ^+^NK1.1^−^DNT cells in B6/*lpr* mice, which partially restored the proportional distribution of TCRγδ^+^DNT and DN‐NKT subpopulations within CD3^+^DNT compartments.

### 3.2 *Egr2* Deletion in B6/*Lpr* Mice Increases IFNγ and IL‐10, but Not IL‐17 Production in TCRβ ^+^ DNT Cells

**FIGURE 2:**
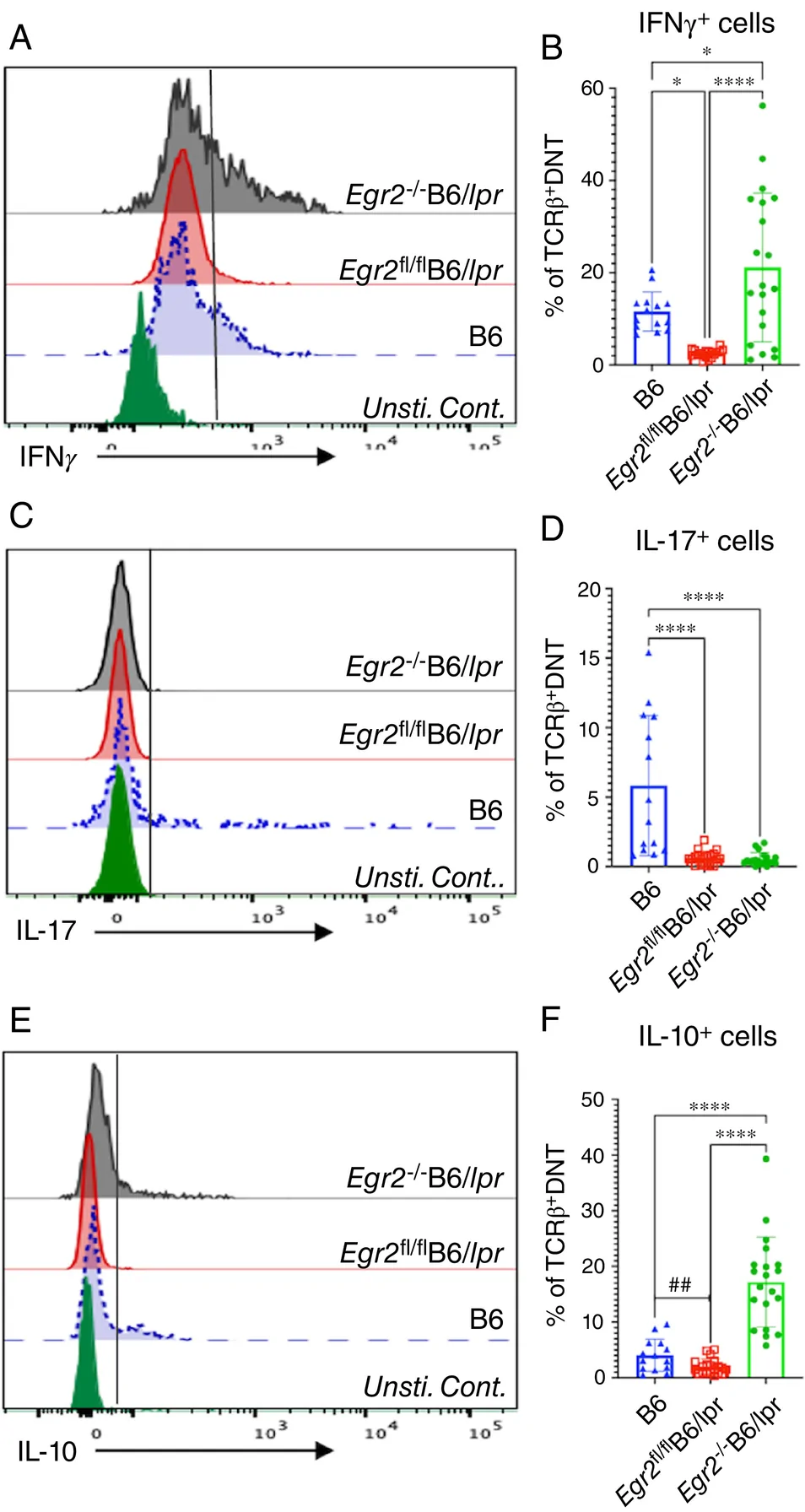
*Egr2* deletion in B6/*lpr* mice increases the expression of IFNγ and IL‐10, but not IL‐17 in TCR𝛽^+^DNT cells. Splenocytes from B6 mice (either *Egr2* ^+/+^ or *Egr2* ^fl/fl^) at 11–36 weeks old, *Egr2* ^fl/fl^B6/*lpr* and *Egr2* ^−/−^B6/*lpr* mice at 15–30 weeks old (*n* ≥ 14 each) were stimulated with PMA plus ionomycin and GolgiPlug for 5 h, and then collected for flow analysis of intracellular cytokines in the gated TCR𝛽^+^DNT cells. The cells without stimulation served as the negative biological control (Unsti. Cont.). Both females and males were included in the assay. (A–F) Representative flow histogram and summary graphs show the percentage of IFNγ^+^ (A, B), IL‐17^+^ (C, D) and IL‐10^+^ (E, F) cells in the gated TCR𝛽^+^DNT cells. Data were obtained from four independent experiments. One‐way ANOVA (displayed with * with brackets) and/or unpaired Student's *t* tests (displayed with # with line) were performed. *p < 0.05; ##*p* < 0.01; and ****p < 0.0001.

The TCRβ^+^DNT cells have been linked to lupus pathogenesis since they produce inflammatory cytokines IFNγ and IL‐17 [30, 31, 32]. Surprisingly, we found that compared to B6 mice, TCRβ^+^DNT cells of B6/*lpr* mice produced lower levels of IFNγ and IL‐17 (Figure 2A–D and Figure S1). *Egr2* deletion in B6/*lpr* mice significantly increased IFNγ but had no effect on IL‐17 expression in the TCRβ^+^DNT cells (Figure 2A–D). TCRβ^+^DNT cells have also been reported to play an immune regulatory role by producing IL‐10 in different studies [4, 5]. Compared to B6, IL‐10 expression was decreased in the TCRβ^+^DNT cells of B6/*lpr* mice. *Egr2* deletion in B6/*lpr* mice significantly increased IL‐10 expression in the TCRβ^+^DNT cells (Figure 2E,F). Given that TCRβ^+^DNT cells of B6 mice comprised DN‐NKT (Figure 1G), we compared the cytokine expression in the gated TCRβ^+^NK1.1^−^ DNT cells of B6 and B6/*lpr* mice. IFNγ, IL‐10, and IL‐17 expression were all significantly lower in TCRβ^+^NK1.1^−^ DNT cells of B6/*lpr* mice (Figure S2). This suggests that the higher cytokine expression in B6 TCRβ^+^DNT cells was not due to the comprisal of DN‐NKT cells.

### 3.3 *Egr2* Deletion in B6/*Lpr* Mice Suppressed IL‐17 in TCRγδ ^+^ DNT Cells, the Major IL‐17‐ Expressing CD3 ^+^ DNT Cells in B6/*Lpr* Mice

**FIGURE 3:**
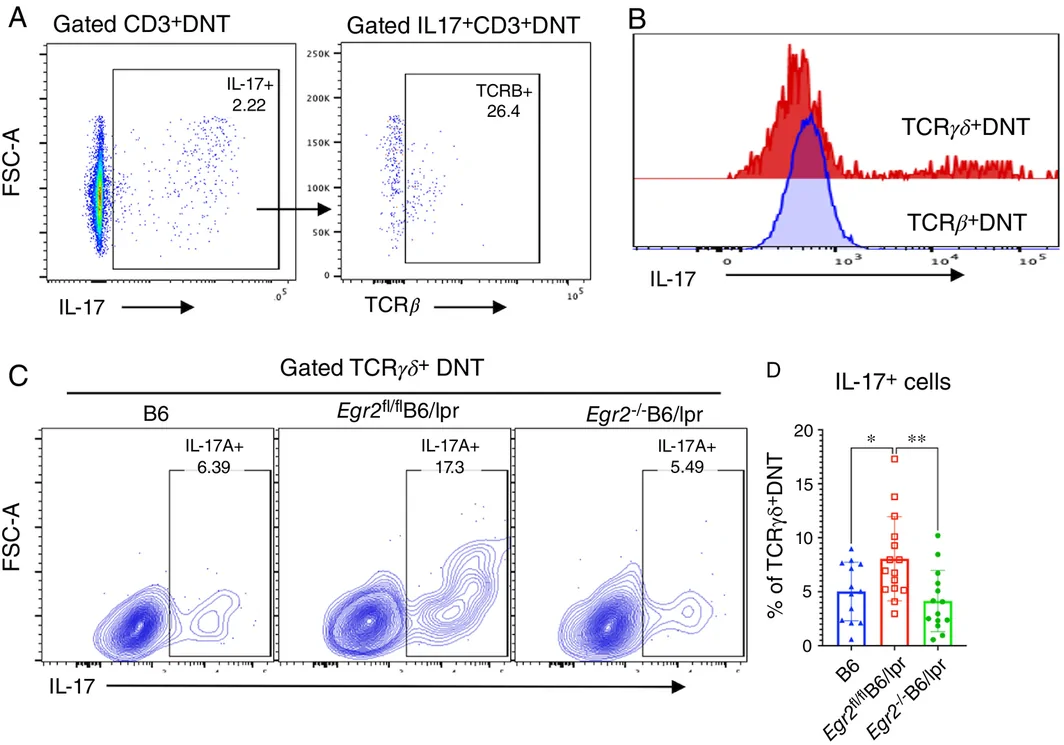
*Egr2* deletion in B6/*lpr* mice suppresses IL‐17 expression in TCRγδ^+^ DNT cells, which are the major IL‐17‐expressing DNT cells in B6/*lpr* mice. Splenocytes from B6 mice (either *Egr2* ^+/+^ or *Egr2* ^fl/fl^) at 11–30 weeks old, *Egr2* ^fl/fl^B6/*lpr* and *Egr2* ^−/−^B6/*lpr* mice at 18–24 weeks old (*n* ≥ 13 each) were stimulated with PMA plus ionomycin and GolgiPlug for 5 h, and then subjected to flow analysis of IL‐17 in the gated cells. Both females and males were included in the assay. (A) Representative flow plots show the percentage of TCR𝛽^+^cells in the gated IL‐17^+^CD3^+^DNT cells of *Egr2* ^fl/fl^B6/*lpr* mice. (B) Representative flow histogram shows IL‐17 expression in the gated TCRγδ^+^ DNT and TCR𝛽^+^DNT cells of *Egr2* ^fl/fl^B6/*lpr* mice. (C, D) Representative flow plots and summary graph show the percentage of IL‐17^+^ cells in the gated TCRγδ^+^DNT cells of B6, *Egr2* ^fl/fl^B6/*lpr* and *Egr2* ^−/−^B6/*lpr* mice. Data were obtained from three independent experiments. One‐way ANOVA (displayed with * with brackets) was performed. *p < 0.05; **p < 0.01.

While most of the IFNγ‐ and IL‐10‐expressing CD3^+^DNT cells were TCRβ^+^ cells (data not shown), the majority of the IL‐17‐expressing CD3^+^DNT cells in B6*/lpr* mice were TCRβ^−^ cells (Figure 3A). Compared to TCRβ^+^DNT cells, there was a higher IL‐17 expression in TCRγδ^+^DNT cells of B6/*lpr* mice (Figure 3B). Compared to B6 mice, there was a significant increase of IL‐17 expression in the TCRγδ^+^DNT cells of B6/*lpr* mice (Figure 3C,D). *Egr2* deletion significantly reduced IL‐17 expression in TCRγδ^+^DNT cells of B6/*lpr* mice (Figure 3C,D).

### 3.4 *Egr2* Deletion in B6/*Lpr* Mice Suppresses CD138 Expression in TCRβ ^+^ DNT Cells

**FIGURE 4:**
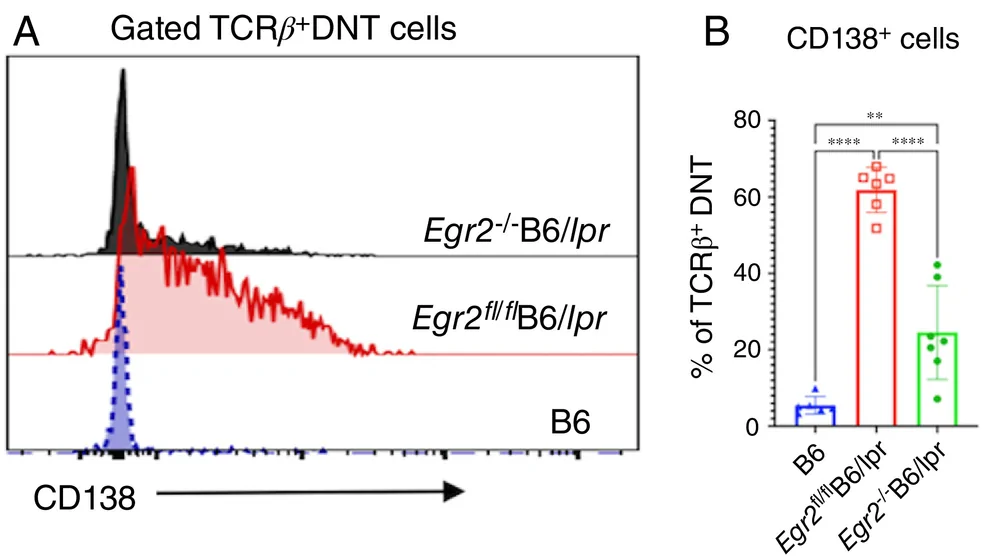
*Egr2* deletion in B6/*lpr* mice suppresses CD138^+^DNT cells, a dominant DNT subpopulation in *lpr* mice. Splenocytes were isolated from B6 mice (either *Egr2* ^+/+^ or *Egr2* ^fl/fl^) at 11–16 weeks old, *Egr2* ^fl/fl^B6/*lpr* and *Egr2* ^−/−^B6/*lpr* mice at 18–22 weeks old (*n* ≥ 6) for flow cytometry. Both male and female mice were included in the assay. Representative flow histogram (A) and summary graph (B) show the percentage of CD138^+^ cells in the gated TCRβ^+^ DNT cells of the experimental mice. Data were obtained from two independent experiments. One‐way ANOVA (displayed with * with brackets) was performed. **p < 0.01; ****p < 0.0001.

CD138‐expressing T (CD138^+^ T) cells, of which the majority are CD4^−^CD8^−^ DNT cells, are highly expanded in MRL/*lpr* mice and play a critical role in promoting autoantibody progression and disease progression [33, 34]. Consistently, we found that most TCRβ^+^DNT cells of B6/*lpr* mice were CD138^+^ cells while the majority of B6 TCRβ^+^DNT cells were CD138^−^. *Egr2* deletion in B6/*lpr* mice significantly reduced CD138 expression in the gated TCRβ^+^DNT cells (Figure 4A,B, Figure S3).

### 3.5 scRNA‐Seq Analysis Reveals EGR2‐Mediated Regulation of DNT Cell Heterogeneity and Function

**FIGURE 5:**
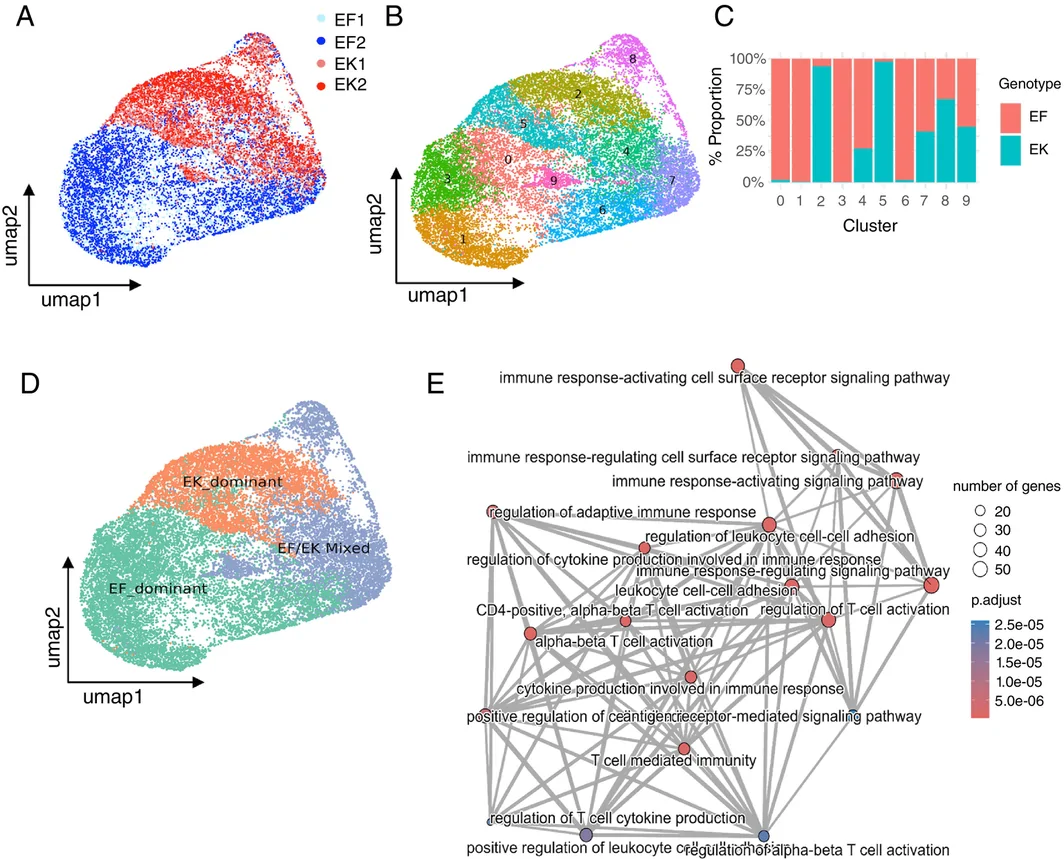
*Egr2* deletion alters the distribution of different DNT clusters in B6/*lpr* mice. CD3^+^DNT cells were sorted from the splenocytes of female *Egr2* ^fl/fl^B6/*lpr* (EF) and *Egr2* ^−/−^B6/*lpr* (EK) mice at 22–23 weeks old for scRNA‐seq analysis (*n* = 2 each group; each was pooled cells of from two mice). (A) The UMAP plot shows a remarkable transcriptional difference between DNT cells of EF (blue hue) and EK (red hue) samples. (B) The UMAP plot shows that the overall DNT cells of EF and EK samples were segregated into 10 clusters (0–9). (C) Stacked bar graph showing the percentage of DNT cells from EF and EK samples in subcluster 0–9. (D) The UMAP plot shows that the overall DNT cells of EF and EK samples were segregated into three major clusters: EF_dominant, EF/EK Mixed, and EK_dominant clusters. (E) Pathway enrichment analysis with the genes that were significantly upregulated in EK_dominant DNT cluster.

To better understand the regulatory effect of EGR2 on the heterogeneity and function of DNT cells, we performed scRNA‐seq analysis of CD3^+^DNT cells from *Egr2* ^−/−^B6/*lpr* and control *Egr2* ^fl/fl^B6/*lpr* mice. A total of 17 686 single DNT cells passed all quality control procedures and were included for downstream analysis. The UAMP visualisation showed both overlapping and segregation of DNT cells between *Egr2* ^−/−^B6/*lpr* and *Egr2* ^fl/fl^B6/*lpr* mice (Figure 5A), indicating that *Egr2* deletion significantly altered the transcriptional profile CD3^+^DNT cells. The overall population of CD3^+^DNT cells from control and *Egr2* ^−/−^B6/*lpr* mice was further clustered into 10 transcriptionally distinct subclusters, each characterised by a unique composition of DNT cells from control and *Egr2* ^−/−^B6/*lpr* mice (Figure 5B,C). Out of 10 subclusters, 4 of them (cluster 0, 1, 3, and 6) were predominantly DNT cells of *Egr2* ^fl/fl^B6/*lpr* mice while DNT cells from *Egr2* ^−/−^B6/*lpr* mice composed more than 95% of cells in cluster 2 and 5 (Figure 5C). According to the composition of DNT subclusters in different genotypes, the 10 subclusters were merged and grouped into three major clusters: EF_dominant, EK_dominant, and EF/EK Mixed (Figure 5D). The genes that were significantly expressed in EF_dominant DNT cluster and EK_dominant DNT cluster were listed (Tables S1 and S2). The pathway enrichment analysis revealed that the significantly expressed genes in EK‐dominant DNT cluster were involved in multiple immune‐related pathways such as immune response activating pathway, T cell mediated immunity, and regulation of alpha‐beta T cell activation (Figure 5E), suggesting a suppressive role of EGR2 on immune response and activation. The significantly expressed genes in EF‐dominant DNT cluster were enriched in cell growth and protein phosphorylation pathways, which is in accordance with the classical function of EGR2 (Figure S4).

**FIGURE 6:**
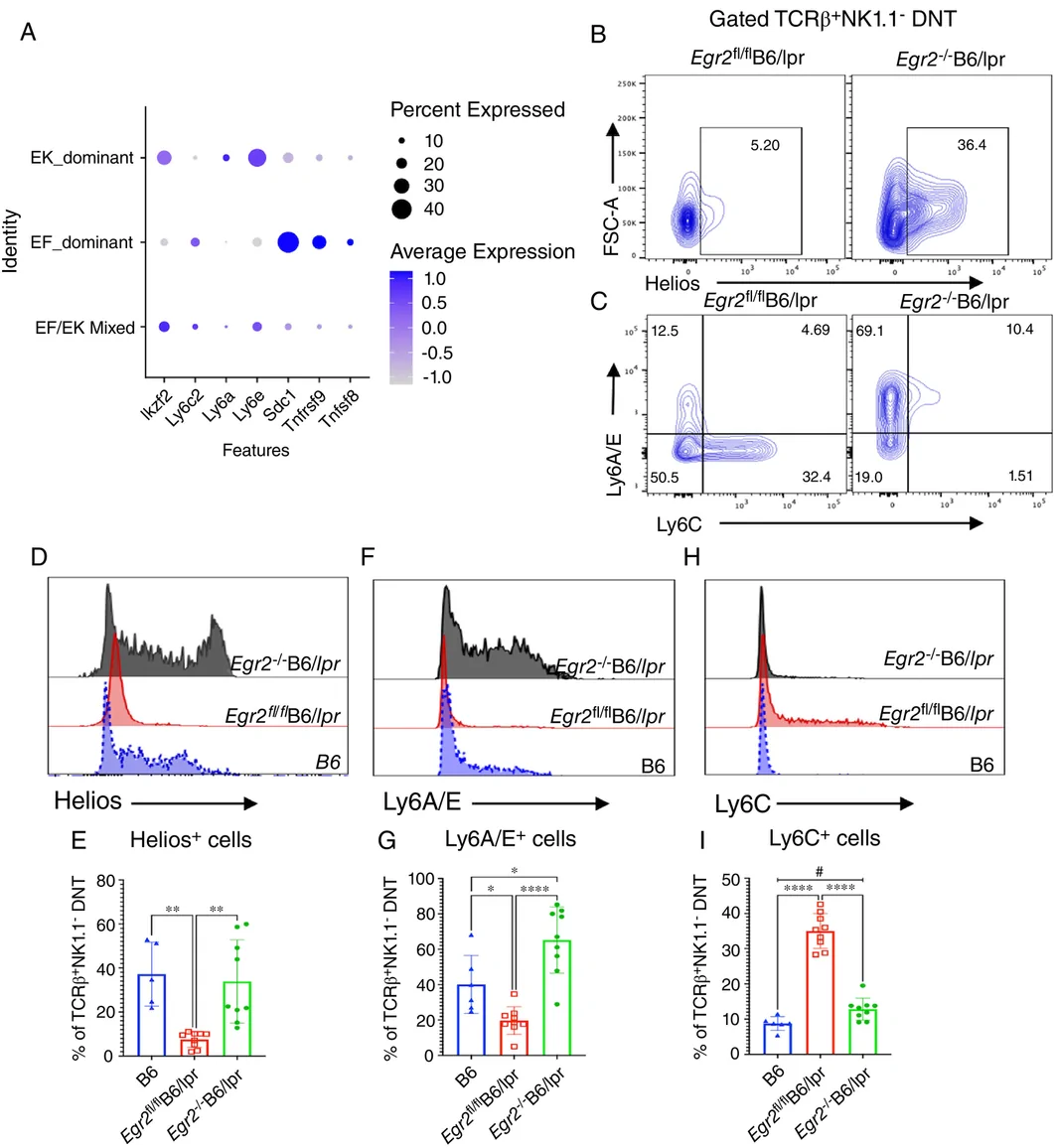
Flow verification of scRNA‐seq data. (A) Dot plots showing the relative expression level and percentage of selected, previously identified marker genes for specific B6 DNT subclusters in EK_dominant, EF_dominant, and EF/EK Mixed DNT cluster. (B–I) Splenocytes were isolated from B6 mice (either *Egr2* ^+/+^ or *Egr2* ^fl/fl^) at 11–30 weeks old, *Egr2* ^fl/fl^B6/*lpr* and *Egr2* ^−/−^B6/*lpr* mice at 18–22 weeks old (*n* ≥ 5) for flow. Both male and female mice were included in the assay. (B and C) Representative flow plots show the expression of Helios (B), Ly6C, and Ly6A/E (C) in the gated TCRβ^+^NK1.1^−^DNT cells of *Egr2* ^fl/fl^B6/*lpr* and *Egr2* ^−/−^B6/*lpr* mice. (D–I) Representative flow histograms and summary graphs show the percentage of Helios^+^ (D, E), Ly6A/E^+^ (F, G), and Ly6C^+^ (H, I) in the gated TCRβ^+^NK1.1^−^DNT cells of B6, *Egr2* ^fl/fl^B6/*lpr*, and *Egr2* ^−/−^B6/*lpr* mice. Data were obtained from two independent experiments. One‐way ANOVA (displayed with * with brackets) and/or unpaired Student's *t* tests (displayed with # with line) were performed. * and #*p* < 0.05; **p < 0.01; ****p < 0.0001.

*Egr2* deletion significantly altered the expression of the genes that were previously identified as key markers for specific nDNT and aDNT subgroups in B6 mice (Figure 6A). *Ikzf2*, a marker gene for both naïve and activated cytotoxic DNT cells, was significantly increased in EK_dominant DNT cells. *Ly6c2*, a key marker gene for all the nDNT clusters except proinflammatory cluster (nDNT1), was significantly reduced in EK_dominant DNT cells. *Ly6a, Tnfsf8*, and *Tnfrsf9* have been reported to be increased during activation of DNT cells from B6 mice [13]. Here, we found that *Tnfsf8* and *Tnfrsf9* were significantly reduced and that *Ly6a/e* were significantly increased in EK_dominant DNT cells (Figure 6A). Consistent with reduced CD138 in the DNT cells of *Egr2* ^−/−^B6/*lpr* (Figure 4), *Sdc1* (encode CD138) was dramatically reduced in EK_dominant DNT cells. The flow analysis verified that Helios (encoded by *Ikzf2*) and Ly6A/E protein expression were significantly increased and Ly6C was significantly decreased in TCRβ^+^NK1.1^−^ DNT cells of *Egr2* ^−/−^B6/*lpr* mice compared to controls (Figure 6B,C). We further showed that compared to B6 mice, Helios and Ly6A/E were significantly decreased, and Ly6C was significantly increased in TCRβ^+^NK1.1^−^ DNT cells of B6/*lpr* (Figure 6D–I and Figure S5). *Egr2* deletion in B6/*lpr* mice reversed the expression changes of these three proteins to make their levels in TCRβ^+^NK1.1^−^ DNT similar to B6 mice (Figure 6D–I).

### 3.6 *Egr2* Deletion in B6/*Lpr* Reduces the Percentage of TCRβ ^+^ Cells, but Increases TCRγδ ^+^ Cells in DN Thymocytes

**FIGURE 7:**
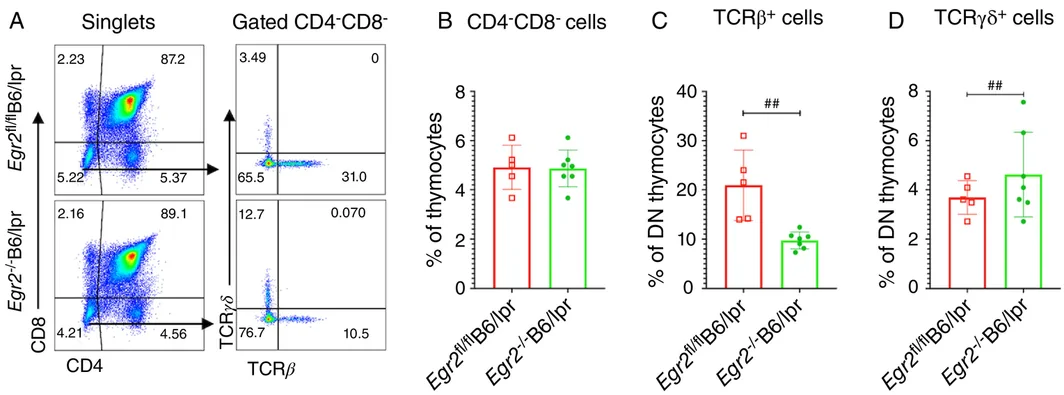
*Egr2* deletion in B6/*lpr* mice reduces the percentage of TCRβ^+^ cells in the gated DN thymocytes. Thymocytes were isolated from *Egr2* ^−/−^B6/*lpr* and control *Egr2* ^fl/fl^B6/*lpr* at 8–12 weeks old mice for flow. Both female and male mice were included in the assay (*n* ≥ 5 each). (A) Representative plots show the gating of TCRβ^+^ and TCRγδ^+^ cells in the gated CD4^−^CD8^−^ DN thymocytes. (B–D) Summary graphs show the percentage DN cells in thymocytes (B), the percentage of TCRβ^+^ cells (C) and TCRγδ^+^ (D) cells in the gated DN thymocytes. Data were obtained from two independent experiments. Unpaired Student's *t* tests (displayed with # with line) were performed. ##*p* < 0.01.

Both thymic origin and extrathymic origin have been proposed for peripheral DNT cells [5, 6]. Here, we found that while *Egr2* deletion did not affect the development of CD4^−^CD8^−^ (DN) thymocytes, it significantly reduced the percentage of TCRβ^+^ cells and increased the percentage of TCRγδ^+^ cells in the gated DN thymocytes (Figure 7A–D). This is consistent with the finding of reduced TCRβ^+^ cells and increased TCRγδ^+^ cells in the gated splenic CD3^+^DNT cells (Figure 1).

### 3.7 Deleting *Egr2* Suppresses the Development of DNT Cells From In Vitro Cultured CD8 ^+^ T Cells

**FIGURE 8:**
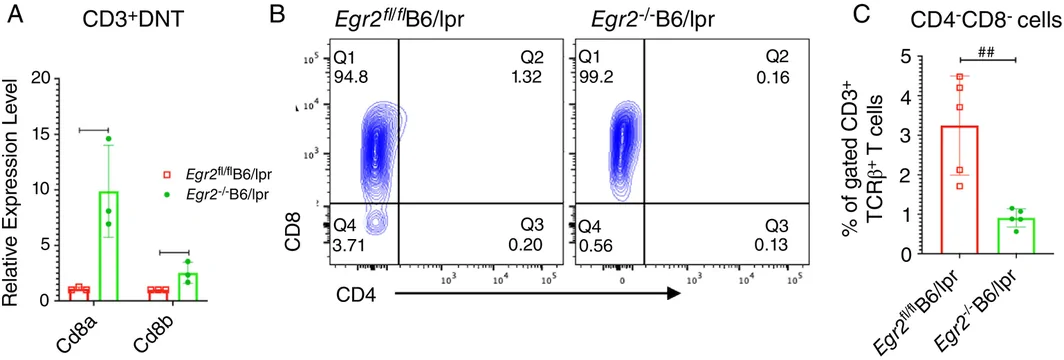
Deleting *Egr2* suppresses the development of DN T cells from in vitro cultured CD8^+^ T cells from B6/*lpr* mice. CD3^+^DNT and CD3^+^CD4^−^CD8^+^ single positive (SP) T cells were sorted from splenocytes of female *Egr2* ^−/−^B6/*lpr* and *Egr2* ^fl/fl^B6/*lpr* mice at 16–20 weeks old. (A) RT‐qPCR analysis of *Cd8a* and *Cd8b* gene expression in the sorted CD3^+^DNT cells (*n* = 3 each, each was the pooled cells from two mice). (B, C) The sorted CD8^+^SP T cells from *Egr2* ^fl/fl^B6/*lpr* and *Egr2* ^−/−^B6/*lpr* mice were cultured with anti‐CD3/anti‐CD28 stimulation for 5 days prior to flow analysis. Representative flow plots (B) and summary graph (C) show the percentage of CD4^−^CD8^−^ cells in the gated CD3^+^TCRβ^+^ cells of the cultured CD8^+^ T cells (*n* = 5 each). Data were obtained from two independent experiments. Unpaired Student's *t* tests (displayed with # with line) were performed. #*p* < 0.05; ##*p* < 0.01.

Current evidence strongly suggests that the peripheral DNT cells in human and murine lupus are mostly derived from the activated CD8^+^ T cells by transcriptional and epigenetic silencing of the *Cd8* gene [6, 26, 35, 36, 37]. In certain conditions, DNT cells may regain CD8 expression, displaying a plasticity between DNT cells and CD8^+^ T cells [5, 36]. Interestingly, we found that *Cd8a*/*Cd8b* expression in the CD3^+^DNT cells of *Egr2* ^−/−^B6/*lpr* mice was significantly increased compared to controls (Figure 8A). Furthermore, the percentage of CD4^−^CD8^−^ T cells in in vitro activated CD8^+^ T cells from *Egr2* ^−/−^B6/*lpr* mice was significantly lower than that from controls (Figure 8B). We did not observe an obvious DNT cell formation from the cultured CD4^+^T cells from either control or *Egr2* ^−/−^B6/*lpr* mice (Data not shown).

## 4 Discussion

The expansion of TCRβ^+^DNT cells in various autoimmune diseases, especially in SLE, is now well acknowledged [4, 5, 6]. The origin and function of these disease‐related TCRβ^+^DNT cells remain unclear. It is likely that at the diseased stage, the TCRβ^+^DNT cells are phenotypically and functionally different compared to the homeostatic state. In this study, we demonstrated that conditionally deleting *Egr2* in B6/*lpr* mice not only dramatically reduced TCRβ^+^DNT cells but also altered the phenotypes and functions of TCRβ^+^DNT cells (Figures 1, 2, 4, 5, 6). This is the first study to show that EGR2 plays a central role in regulating the expansion and function of TCRβ^+^DNT cells in *lpr* lupus mice.

Previous studies have demonstrated that TCRβ^+^DNT cells play a pro‐inflammatory role in lupus by producing inflammatory cytokines IFNγ and IL‐17 [30, 31, 32]. IL‐10 has been reported to be increased in human and murine lupus [38, 39]. Here, we surprisingly found that compared to B6, IFNγ, IL‐17, and IL‐10 expression were all significantly decreased in TCRβ^+^DNT cells of B6/*lpr* mice. *Egr2* deletion in B6/*lpr* mice significantly increased IFNγ and IL‐10 expression, but not IL‐17 in TCRβ^+^DNT cells. The CD138 (Syndecan 1), a known marker for antibody‐producing plasma cells and plasmablasts, is also expressed on T cells in the context of autoimmune disease, particularly in *lpr* lupus mice [33, 34]. The majority of the CD138^+^ T cells of *lpr* mice are DNT cells. Compared to CD138^−^ T cells, CD138^+^DNT cells were less responsive to T cell stimulation and produced less cytokines such as IFNγ and IL‐17, and were more potent in helping antibody production from autoreactive B cells with the presence of autoantigen [33]. Therefore, the reduced cytokine expression in TCRβ^+^DNT cells of B6/*lpr* mice is likely due to the significant CD138 expression (Figure 4). With the depletion of CD138^+^DNT cells, there was a significant increase in the expression of cytokines in TCRβ^+^DNT cells (Figure 2) with a concomitant significant decrease of autoantibodies in *Egr2* ^−/−^B6/*lpr* mice [20].

It has been suggested that TCRγδ^+^DNT cells are not involved in the increase of IL‐17 in human patients with lupus [30]. However, we found that the major source of IL‐17‐producing DNT cells in B6/*lpr* mice was TCRγδ^+^DNT‐cell (Figure 3B). *Egr2* deletion suppressed IL‐17 in the TCRγδ^+^DNT cells (Figure 3C,D), which is consistent with our previous finding of the positive role of EGR2 on IL‐17 production in B6/*lpr* mice [20]. Whether and how IL‐17‐expressing TCRγδ^+^DNT cells are involved in the pathogenesis of B6/*lpr* lupus mice remains to be investigated.

With scRNA‐seq analysis, we demonstrated a remarkable difference in the transcriptional profile of DNT cells from *Egr2* ^−/−^B6/*lpr* and *Egr2* ^fl/fl^B6/*lpr* mice. In a previous study using DNT cells from B6 mice, Helios has been identified as the most important transcription factor for defining cytotoxic DNT cells [13]. Ly6C (mainly Ly6C2) has been identified as a key marker for non‐proinflammatory DNT clusters, while Ly6A/E is highly expressed in proinflammatory DNT cells [13]. Here, we found that Helios and Ly6A/E were significantly reduced, and Ly6C was significantly increased in TCRβ^+^NK1.1^−^DNT of B6/*lpr* mice compared to B6 mice, suggesting a potential reduction of both cytotoxic DNTs and proinflammatory DNTs in B6/*lpr* mice. This explains the reduction of IFNγ (a mediator of cytotoxic DNT function) and IL‐17 (a mediator of proinflammatory DNT) in TCRβ^+^NK1.1^−^DNT cells of B6/*lpr* mice. *Egr2* deletion in B6/*lpr* mice likely promoted both cytotoxic DNT and proinflammatory DNT cells with a significant increase of Helios and Ly6A/E and a decrease of Ly6C in TCRβ^+^NK1.1^−^DNT cells. The increase of cytotoxic DNTs is consistent with the increased IFNγ expression in the TCRβ^+^DNT of *Egr2* ^−/−^B6/*lpr* mice. However, we did not observe an increase in IL‐17 in TCRβ^+^DNT of *Egr2* ^−/−^B6/*lpr* mice. This may be because the major source of IL‐17‐expressing DNT cells in B6/*lpr* mice was TCRγδ^+^DNT‐cell (Figure 3).

In this study, we also investigated the mechanism by which EGR2 regulates DNT‐cell development. The loss of marginal zone macrophages (MZMs) has been linked to DNT development driven by autoreactive CD8^+^ T cells in lupus [37]. However, *Egr2* deletion did not promote MZMs to suppress DNT cell development in B6/*lpr* mice (Figure S6). Interestingly, we found that *Egr2* deletion in B6/*lpr* mice reduced the percentage of TCRβ^+^DN thymocytes (Figure 7), increased *Cb8a/b* expression in DNT cells, and suppressed DNT development from in vitro activated CD8^+^ T cells (Figure 8). These data suggest that EGR2 may regulate TCRβ^+^DNT‐cell development at both T cell development stages in the thymus and in peripheral lymphoid organs from activated CD8^+^ T cells, although the mechanism remains elusive. Given the emerging epigenetic regulatory role of EGR2 [40, 41], our future studies will investigate whether and how EGR2 epigenetically silences *Cd8* in B6/*lpr* CD8^+^ T cells to expand peripheral DNT cells.

The mutation of the *Fas* gene in *lpr* mice prevents Fas ligand (FasL)/Fas‐mediated apoptosis of activated lymphocytes, which leads to the massive accumulation of DNT cells among other lymphocytes and consequently lymphadenopathy in *lpr* mice [24, 42, 43]. EGR2 has been shown to induce *FasL* expression in activated T cells via binding to a specific Fas ligand regulatory element (FLRE) in the *FasL* gene promoter, suggesting a positive role of EGR2 in FasL/Fas‐mediated apoptosis of activated lymphocytes [44, 45]. In addition, EGR2 induces PTEN‐mediated apoptosis in cancer cells by directly upregulating proapoptotic Bcl‐2 family members [46]. Nevertheless, neither overexpression nor knockout of *Egr2* gene in vivo in normal B6 mice affected *FasL* gene expression or thymocyte apoptosis [47]. Here, we found that *Egr2* deletion did not affect anti‐CD3‐induced apoptosis in DNT cells or in CD4^+^ and CD8^+^ T cells (Figure S7). Given the potential positive role of EGR2 in apoptosis and the intrinsic defect of the FasL/Fas‐mediated apoptosis pathway in *lpr* mice, it is unlikely that *Egr2* deletion suppresses DNT cell development by inducing DNT cell apoptosis via FasL/Fas‐mediated apoptosis or other cell death pathways.

By applying the markers identified in B6 DNT cells, we demonstrated the changes in the composition of cytotoxic DNT (defined by Helios^+^) and pro‐inflammatory DNT (defined by Ly6A/E^+^ and Ly6C^−^) in B6, B6/*lpr*, and *Egr2* ^−/−^B6/*lpr* mice. However, the direct correlation of cytokine expression with specific DNT subgroups in TCRβ^+^DNT cells of B6/*lpr* mice was not defined in our current studies. Considering the autoinflammatory state of B6/*lpr* lupus mice, it is likely that even without stimulation, the freshly‐isolated DNT cells of *B6/lpr* mice comprise both naïve and aDNTs. Xu, et al. have reported that the expression of aDNT markers (CD30, CD137, and CD153) was significantly higher in the DNT of MRL/*lpr* mice compared to B6 mice [48]. It is important and necessary to integrate deep machine learning methods into the analysis of scRNA‐seq data with DNT cells from *Egr2* ^−/−^B6/*lpr*, *Egr2* ^fl/fl^B6/*lpr*, and B6 mice together to refine scRNA‐seq clustering and to determine the phenotypic and functional differences and/or similarity of DNT cells among B6, B6/*lpr*, and *Egr2* ^−/−^B6/*lpr*. Despite the limitations, our studies clearly revealed that EGR2 is critical for the accumulation of DNT cells with phenotypic and functional abnormalities in *lpr* lupus mice and that *Egr2* deletion in B6/*lpr* mice tends to correct this abnormality. Our data provide a novel insight into the molecular regulation of DNT cell development and function in lupus.

## Author Contributions

R.D., S.A.A., and C.M.R. conceived the study and secured the funds for the research. R.D. designed and performed experiments with assistance from Z.W. and B.H. Z.W. performed the scRNA‐seq data analysis. R.D. and S.A.A. drafted, wrote, and finalised the manuscript. C.M.R. critically reviewed and edited the manuscript. All authors have read and approved the final manuscript for publication.

## Funding

This study was supported by the Edward Via College of Osteopathic Medicine (VCOM)‐Virginia‐Maryland College of Veterinary Medicine (VMCVM) One Health Research Seed grant (#461722) and the VMCVM Intramural Research Competition (IRC) Grant (#180472).

## Ethics Statement

All animal use and experimental procedures were conducted with the approval and oversight of the Institutional Animal Care and Use Committee (IACUC) of Virginia Tech under protocols 21–255‐CVM and 22–115‐CVM.

## Consent

The authors have nothing to report.

## Conflicts of Interest

The authors declare no conflicts of interest.

## Supporting information


**Figure S1:** *Egr2* deletion in B6/*lpr* mice increases the expression of IFNγ and IL‐10, but not IL‐17 in TCR𝛽^+^DNT cells. Splenocytes from B6 mice (either *Egr2* ^+/+^ or *Egr2* ^fl/fl^) at 11–36 weeks old), *Egr2* ^fl/fl^B6/*lpr* and *Egr2* ^−/−^B6/*lpr* mice at 15–30 weeks old (*n* ≥ 14 each) were stimulated with PMA plus ionomycin and GolgiPlug for 5 h and then collected for flow analysis of intracellular cytokines. The cells without stimulation served as the negative biological control (Unstimulated control). Both females and males were included in the assay. (A) Flow cytometry gating strategy for the identification of TCR𝛽^+^DNT cells. (B) Representative flow dot plots show the gating and percentage of IFNγ^+^ (upper panel), IL‐17^+^ (middle panel) and IL‐10^+^ (lower panel) cells in the gated TCR𝛽^+^DNT cells of B6, *Egr2* ^fl/fl^B6/*lpr*, and *Egr2* ^−/−^B6/*lpr* mice.
**Figure S2:** The expression of IFN𝛾, IL‐10 and IL‐17 in TCR𝛽^+^NK1.1^−^DNT cells of B6/*lpr* mice is significantly lower than B6 mice. Splenocytes from B6 mice (*Egr2* ^fl/fl^) at 25–27 weeks old) and B6/*lpr* (*Egr2* ^fl/fl^B6/*lpr*) at 20–30 weeks old (*n* ≥ 5) were stimulated with PMA plus ionomycin and GolgiPlug for 5 h, and then collected for flow analysis of intracellular cytokines in the gated TCR𝛽^+^NK1.1^−^DNT cells. Both females and males were included in the assay. (A–C) Summary graphs show the percentage of IFNγ^+^ (A), IL‐110^+^ (B) and IL‐17^+^ (C) cells in the gated TCR𝛽^+^NK1.1^−^DNT cells. Unpaired Student's *t* tests were performed (displayed with # with line). # *p* < 0.05; and ## *p* < 0.01.
**Figure S3:** *Egr2* deletion in B6/*lpr* mice suppresses CD138^+^DNT cells, a dominant DNT subpopulation in *lpr* mice. Splenocytes were isolated from B6 mice (either *Egr2* ^+/+^ or *Egr2* ^fl/fl^) at 11–16 weeks old, *Egr2* ^fl/fl^B6/*lpr* and *Egr2* ^−/−^B6/*lpr* mice at 18–22 weeks old (*n* ≥ 6) for flow cytometry. Both male and female mice were included in the assay. Representative flow dot plots show the gating and percentage of CD138^+^ cells in the gated TCRβ^+^ DNT cells of B6, *Egr2* ^fl/fl^B6/*lpr*, and *Egr2* ^−/−^B6/*lpr* mice.
**Figure S4:** Pathway enrichment analysis with the genes that were significantly upregulated in EF_dominant DNT cells.
**Figure S5:** *Egr2* deletion in B6/*lpr* mice normalised the expression of Helios, Ly6A/E, and Ly6C in TCRβ^+^NK1.1^−^DNT cells to levels comparable to those observed in B6 mice. Splenocytes were isolated from B6 mice (either *Egr2* ^+/+^ or *Egr2* ^fl/fl^) at 11–30 weeks old, *Egr2* ^fl/fl^B6/*lpr* and *Egr2* ^−/−^B6/*lpr* mice at 18–22 weeks old (*n* ≥ 5) for flow. Both male and female mice were included in the assay. (A) Flow cytometry gating strategy for the identification of TCRβ^+^NK1.1^−^DNT cells. (B) Representative flow dot plots show the gating and percentage of Helios^+^ (upper panel), Ly6A/E^+^ (middle panel) and Ly6C^+^ (lower panel) in the gated TCRβ^+^NK1.1^−^DNT cells of B6, *Egr2* ^fl/fl^B6/*lpr*, and *Egr2* ^−/−^B6/*lpr* mice.
**Figure S6:** *Egr2* deletion in B6/*lpr* mice has no significant effect on the percentage of marginal zone macrophage (MZM), which is reduced in B6/*lpr* mice compared to B6 mice. Splenocytes were prepared from female B6 mice (*Egr2* ^fl/fl^) at 36–40 weeks old, female *Egr2* ^fl/fl^B6/*lpr* and *Egr2* ^−/−^B6/*lpr* mice at 15–24 weeks old (*n* ≥ 5) for flow cytometry. (A) Representative flow plots show the gating and percentage of CD11b^int^F4/80^−^ cells in splenocytes of B6, *Egr2* ^fl/fl^B6/*lpr* and *Egr2* ^−/−^B6/*lpr* mice. (B) Summary graph shows that *Egr2* deletion in B6/*lpr* mice further increased the percentage of CD11b^int^F4/80^−^ cells in splenocytes, which was increased in B6/*lpr* mice compared to B6 mice. (C) Representative flow plots show the further gating of MHCII^−^SIGN‐R1^+^ in the gated CD11b^int^F4/80^−^ cells of B6, *Egr2* ^fl/fl^B6/*lpr* and *Egr2* ^−/−^B6/*lpr* mice. (D) Summary graph shows that *Egr2* deletion in B6/*lpr* mice did not change the percentage of MHCII^−^SIGN‐R1^+^ in the gated CD11b^int^F4/80^−^ cells of B6/*lpr* mice, which was significantly reduced when compared to B6 mice. (E) Summary graph shows that the percentage of MZM cells (CD11b^int^F4/80^−^MHCII^−^SIGN‐R1^+^) in splenocytes was significantly reduced in B6/*lpr* mice compared to B6 mice and that *Egr2* deletion in B6/*lpr* mice had no effect on splenic MZM development. One‐way ANOVA (displayed with * with brackets) and/or unpaired Student's *t* tests (displayed with # with line) were performed. #*p* < 0.05; **and ##*p* < 0.01; ***p < 0.001; ****p < 0.0001.
**Figure S7:** *Egr2* deletion in B6/*lpr* mice has no significant effect on the apoptosis of T lymphocytes. Splenocytes were prepared from *Egr2* ^fl/fl^B6/*lpr* and *Egr2* ^−/−^B6/*lpr* mice at 27 weeks old (*n* = 4 each) and stimulated with plate‐coated anti‐CD3 (5 μg/mL) for 20 h. The stimulated cells were collected for apoptosis assay with Annexin V Apoptosis Detection Kit with PI (Biolegend, CA). The cells were stained with surface markers prior to the incubation with Annexin V and PI in Annexin V binding buffer. (A) Representative flow plot showed the gating of early apoptotic (Annexin V^+^PI^−^) and late apoptotic (Annexin V^+^PI^+^) cells. There was no obvious difference in the percentages of early and late apoptotic cells in the gated CD3^+^DNT cells (top panel), CD4^+^ T (middle panel) and CD8^+^ T (bottom panel) cells between *Egr2* ^fl/fl^B6/*lpr* and *Egr2* ^−/−^B6/*lpr* mice. (B–D) Representative flow histograms and summary graphs showed that *Egr2* deletion in B6/*lpr* had no obvious effect on the percentage of Annexin V^+^ cells in the gated CD3^+^DNT cells (B), CD4^+^ (C) and CD8^+^ T (D) cells.

**Table S1:** The list of genes upregulated in EF_dominant DNT cluster.

**Table S2:** The list of genes upregulated in EK_dominant DNT cluster.

## Data Availability

The original data that support the findings of this study are included in the article/Supporting Information [Link], [Link], [Link]. Further inquiries can be directed to the corresponding authors.
