# Supplementary material for: The Transcription Factor EGR2 Plays a Central Role in the Expansion and Function of TCRαβ + CD4 − CD8 − Double Negative T Cells in lpr Lupus Mice
Source: Immunology. 2026 Jun 21;179(2):224–35. doi: 10.1111/imm.70162 (PMC13540510; doi:10.1111/imm.70162)
Supplement: Supplementary file 1 [file IMM-179-224-s002.docx]

**Supplemental Figure 1: *Egr2* deletion in B6/*lpr* mice increases the expression of IFNγ and IL-10, but not IL-17 in TCR𝛽^+^DNT cells.** Splenocytes from B6 mice (either *Egr2*^+/+^ or *Egr2*^fl/fl^) at 11-36 weeks old), *Egr2*^fl/fl^B6/*lpr* and *Egr2*^-/-^B6/*lpr* mice at 15-30 weeks old (n≥14 each) were stimulated with PMA plus ionomycin and GolgiPlug for 5 hours and then collected for flow analysis of intracellular cytokines. The cells without stimulation served as the negative biological control (Unstimulated control). Both females and males were included in the assay. **(A)** Flow cytometry gating strategy for the identification of TCR𝛽^+^DNT cells. (**B**) Representative flow dot plots show the gating and percentage of IFNγ^+^ (upper panel), IL-17^+^ (middle panel) and IL-10^+^ (lower panel) cells in the gated TCR𝛽^+^DNT cells of B6, *Egr2*^fl/fl^B6/*lpr,* and *Egr2*^-/-^B6/*lpr* mice.

**Supplemental Figure 2: The expression of IFN𝛾, IL-10 and IL-17 in TCR𝛽^+^NK1.1^-^DNT cells of B6/*lpr* mice is significantly lower than B6 mice.** Splenocytes from B6 mice (*Egr2*^fl/fl^) at 25-27 weeks old) and B6/*lpr* (*Egr2*^fl/fl^B6/*lpr)* at 20-30 weeks old (n≥5) were stimulated with PMA plus ionomycin and GolgiPlug for 5 hours, and then collected for flow analysis of intracellular cytokines in the gated TCR𝛽^+^NK1.1^-^DNT cells. Both females and males were included in the assay. (**A-C**) Summary graphs show the percentage of IFNγ^+^ (**A**), IL-110^+^ (**B**) and IL-17^+^ (**C**) cells in the gated TCR𝛽^+^NK1.1^-^DNT cells**.** Unpaired Student's *t* tests were performed (displayed with # with line). # *P*< 0.05; and ## *P*< 0.01.

**Supplemental Figure 3: *Egr2* deletion in B6/*lpr* mice suppresses CD138^+^DNT cells, a dominant DNT subpopulation in *lpr* mice.** Splenocytes were isolated from B6 mice (either *Egr2*^+/+^ or *Egr2*^fl/fl^) at 11-16 weeks old, *Egr2*^fl/fl^B6/*lpr* and *Egr2*^-/-^B6/*lpr* mice at 18-22 weeks old (n≥6) for flow cytometry. Both male and female mice were included in the assay. Representative flow dot plots show the gating and percentage of CD138^+^ cells in the gated TCRβ^+^ DNT cells of B6, *Egr2*^fl/fl^B6/*lpr,* and *Egr2*^-/-^B6/*lpr* mice.

**Supplemental Figure 4: Pathway enrichment analysis with the genes that were significantly upregulated in EF_dominant DNT cells.**

**Figure 5: *Egr2* deletion in B6/*lpr* mice normalized the expression of Helios, Ly6A/E, and Ly6C in TCRβ^+^NK1.1^-^DNT cells to levels comparable to those observed in B6 mice.** Splenocytes were isolated from B6 mice (either *Egr2*^+/+^ or *Egr2*^fl/fl^) at 11-30 weeks old, *Egr2*^fl/fl^B6/*lpr* and *Egr2*^-/-^B6/*lpr* mice at 18-22 weeks old (n≥5) for flow. Both male and female mice were included in the assay. **(A)** Flow cytometry gating strategy for the identification of TCRβ^+^NK1.1^-^DNT cells. **(B)** Representative flow dot plots show the gating and percentage of Helios^+^ (upper panel), Ly6A/E^+^ (middle panel) and Ly6C^+^ (lower panel) in the gated TCRβ^+^NK1.1^-^DNT cells of B6, *Egr2*^fl/fl^B6/*lpr,* and *Egr2*^-/-^B6/*lpr* mice.

**Supplemental Figure 6: *Egr2* deletion in B6/*lpr* mice has no significant effect on the percentage of marginal zone macrophage (MZM), which is reduced in B6/*lpr* mice compared to B6 mice.** Splenocytes were prepared from female B6 mice (*Egr2*^fl/fl^) at 36-40 weeks old, female *Egr2*^fl/fl^B6/*lpr* and *Egr2*^-/-^B6/*lpr* mice at 15-24 weeks old (n≥5) for flow cytometry. (**A**) Representative flow plots show the gating and percentage of CD11b^int^F4/80^-^ cells in splenocytes of B6, *Egr2*^fl/fl^B6/*lpr* and *Egr2*^-/-^B6/*lpr* mice*.* (**B**) Summary graph shows that *Egr2* deletion in B6/*lpr* mice further increased the percentage of CD11b^int^F4/80^-^ cells in splenocytes, which was increased in B6/*lpr* mice compared to B6 mice. (**C**) Representative flow plots show the further gating of MHCII^-^SIGN-R1^+^ in the gated CD11b^int^F4/80^-^ cells of B6, *Egr2*^fl/fl^B6/*lpr* and *Egr2*^-/-^B6/*lpr* mice. (**D**) Summary graph shows that *Egr2* deletion in B6/*lpr* mice did not change the percentage of MHCII^-^SIGN-R1^+^ in the gated CD11b^int^F4/80^-^ cells of B6/*lpr* mice, which was significantly reduced when compared to B6 mice. (**E**) Summary graph shows that the percentage of MZM cells (CD11b^int^F4/80^-^MHCII^-^SIGN-R1^+^) in splenocytes was significantly reduced in B6/*lpr* mice compared to B6 mice and that *Egr2* deletion in B6/*lpr* mice had no effect on splenic MZM development. One-way ANOVA (displayed with * with brackets) and/or unpaired Student's *t* tests (displayed with # with line) were performed. # *P* < 0.05; ** and ## *P*< 0.01; *** *P* < 0.001; **** *P* < 0.0001.

**Supplemental Figure 7: *Egr2* deletion in B6/*lpr* mice has no significant effect on the apoptosis of T lymphocytes.** Splenocytes were prepared from *Egr2*^fl/fl^B6/*lpr* and *Egr2*^-/-^B6/*lpr* mice at 27 weeks old (n=4 each) and stimulated with plate-coated anti-CD3 (5μg/ml) for 20 hours. The stimulated cells were collected for apoptosis assay with Annexin V Apoptosis Detection Kit with PI (Biolegend, CA). The cells were stained with surface markers prior to the incubation with Annexin V and PI in Annexin V binding buffer. (**A**) Representative flow plot showed the gating of early apoptotic (Annexin V^+^PI^-^) and late apoptotic (Annexin V^+^PI^+^) cells. There was no obvious difference in the percentages of early and late apoptotic cells in the gated CD3^+^DNT cells (top panel), CD4^+^ T (middle panel) and CD8^+^ T (bottom panel) cells between *Egr2*^fl/fl^B6/*lpr* and *Egr2*^-/-^B6/*lpr* mice. **(B-D)** Representative flow histograms and summary graphs showed that *Egr2* deletion in B6/*lpr* had no obvious effect on the percentage of Annexin V^+^ cells in the gated CD3^+^DNT cells (**B**), CD4^+^ (**C**) and CD8^+^ T (**D**) cells.
